# Postmenopausal overweight and breast cancer risk; results from the KARMA cohort

**DOI:** 10.1007/s10549-022-06664-7

**Published:** 2022-08-30

**Authors:** Marie Klintman, Ann H. Rosendahl, Benjamin Randeris, Mikael Eriksson, Kamila Czene, Per Hall, Signe Borgquist

**Affiliations:** 1 Division of Oncology, Department of Clinical Sciences Lund, Skåne University Hospital, Lund University, 221 85 Lund, Sweden; 2 Department of Clinical Epidemiology, Aarhus University, Aarhus, Denmark; 3 Karolinska Institute, Stockholm, Sweden; 4 Department of Oncology, Södersjukhuset, Stockholm, Sweden; 5 Department of Oncology, Aarhus University Hospital, Aarhus University, Aarhus, Denmark

**Keywords:** Overweight, Obesity, BMI, Breast cancer risk, Breast cancer subtypes

## Abstract

**Purpose:**

To study the risk of incident breast cancer and subtype-specific breast cancer in relation to excess body weight in a contemporary Swedish prospective cohort study, The Karolinska Mammography Project for Risk Prediction of Breast Cancer, KARMA.

**Methods:**

A total of 35,412 postmenopausal women attending mammography and included in the KARMA study provided baseline data on body mass index (BMI) and potential confounders. During eight years of follow-up, 822 incident invasive breast cancer cases were identified.

**Results:**

Women with overweight (BMI ≥ 25–< 30 kg/m^2^) constituting 34% of the study cohort had an increased risk of incident breast cancer with an adjusted Hazard Ratio (HR_adj_) 1.19 (95% CI 1.01–1.4). A similar, however, non-significant, association was found for women with obesity (BMI ≥ 30 kg/m^2^) conferring 13% of the cohort, with a HR_adj_ of 1.19 (95% CI 0.94–1.5). Overweight was associated with risk of node-negative disease (HR_adj_ 1.29, 95% CI 1.06–1.58), whereas obesity was associated with node-positive disease (HR_adj_ 1.64, 95% CI 1.09–2.48). Both overweight and obesity were associated with risk of estrogen receptor positive (ER+) disease (HR_adj_ 1.20, 95% CI 1.00–1.44 and HR_adj_ 1.33, 95% CI 1.03–1.71, respectively), and low-grade tumors (HR_adj_ 1.25, 95% CI 1.02–1.54, and HR_adj_ 1.40, 95% CI 1.05–1.86, respectively). Finally, obesity was associated with ER+HER2 negative disease (HR_adj_ 1.37, 95% CI 1.05–1.78) and similarly luminal A tumors (HR_adj_ 1.43, 95% CI 1.02–2.01).

**Conclusion:**

Overweight and obesity are associated with an increased risk of developing breast cancer, specifically ER+, low-grade, and for obesity, node-positive, high-risk breast cancer indicating a further need for risk communication and preventive programs.

## Introduction

According to the World Health Organization (WHO) overweight and obesity has tripled since 1975 worldwide, and in 2016, 1.6 billion adults were classified as overweight (BMI ≥ 25 kg/m^2^), out of which 650 million were obese (BMI ≥ 30 kg/m^2^). Obesity is classified as a chronic, but preventable disease [1] associated with higher risks of developing several types of cancer including breast cancer, but also a higher cancer mortality [2, 3]. The molecular mechanisms underlying the higher cancer incidence and cancer mortality associated with overweight and obesity are not yet fully understood. However, studies have identified associations with tumor angiogenesis, and an increase in pro-inflammatory cytokines promoting tumor growth, invasion, and metastatic potential [3]. For breast cancer, most earlier studies have found an association between obesity and risk of postmenopausal breast cancer [4–7], even though recent publications have modified the picture and suggested that the risk may be limited to women with adulthood overweight, and especially postmenopausal weight-gain, and not to women who have been overweight from childhood, as childhood overweight seems to exert a protective effect against breast cancer risk [8]. The association between specific tumor types and in overweight/obese women is, however, less clarified. In postmenopausal women, the majority of studies find a positive association between overweight and risk of estrogen receptor positive (ER+)/progesterone receptor positive (PR+) breast cancer [5, 9], especially in women who have not used hormone replacement therapy (HRT) [4, 10–13], whereas results are conflicting regarding the risk of triple negative breast cancer (TNBC) [4, 5, 12, 14]. Lastly, overweight and obesity at the time of diagnosis has been associated with unfavorable prognostic variables such as larger tumor size and nodal status [5, 15] and a worse prognosis [5, 16].

In this study, we aim at studying the risk of developing postmenopausal breast cancer, subtype-specific breast cancer, as well as associations with known prognostic variables in relation to adiposity, in a contemporary, modern, prospective Swedish cohort study, KARMA (KArolinska Mammography Project for Risk Prediction of Breast Cancer), consisting of more than 70,000 women included from 2011 to 2013.

## Methods

### Study population

**Fig. 1:**
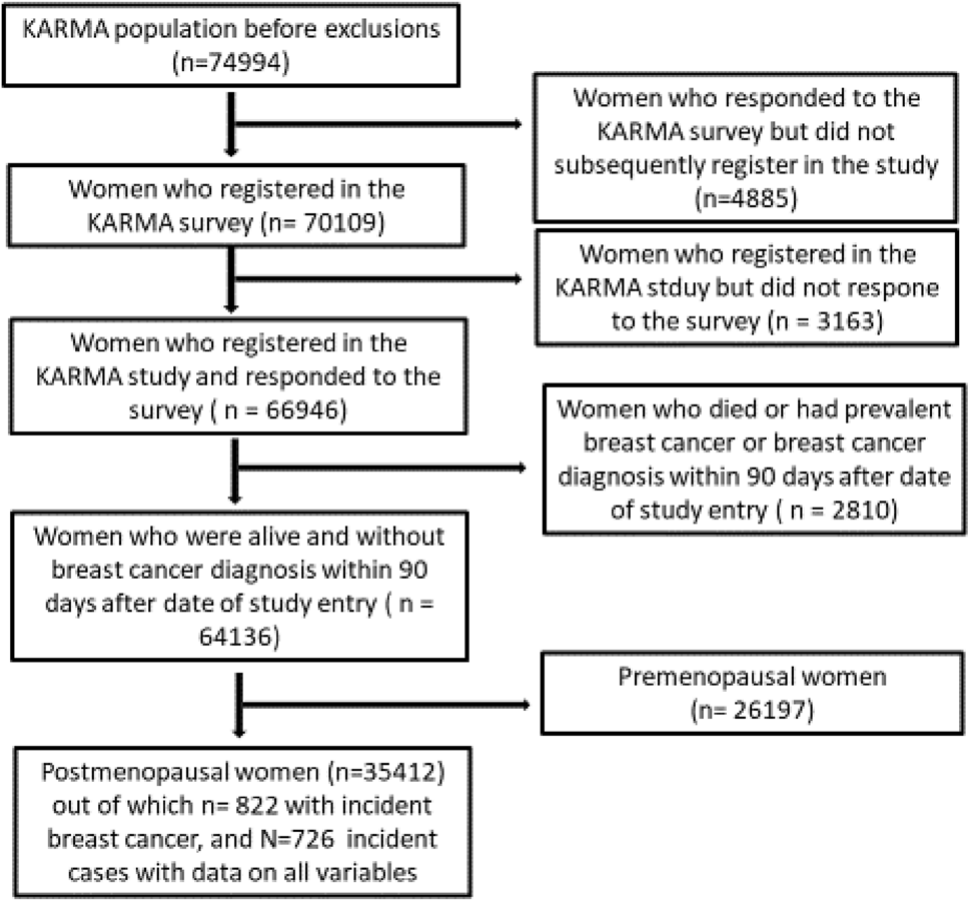
CONSORT flow diagram

The study population consists of 74,877 Swedish women included in the KARMA Cohort (the KARolinska MAmmography Project for Risk Prediction of Breast Cancer, http:/karmastudy.org) [17], a study initiated with the ultimate goal of reducing the incidence and mortality in breast cancer by focusing on individualized prevention and screening. Between January 2011 and March 2013, all women undergoing clinical or screening mammography at four hospitals in Sweden (Södersjukhuset, Stockholm, Helsingborg Hospital, Skåne University Hospital, and Landskrona Hospital), were invited to participate in the study. An informed consent was signed, and at inclusion the participants answered detailed web-based life-style questionnaires and donated blood. Permission for linkage to Swedish national Patient-, Prescription, Cancer-, and Cause of Death registers with access to information on tumor characteristics and treatment data (the INCA and NKBC Register [18]), prescriptions (the Swedish Prescription Register [19]), cancer incidence (the Cancer Register [20]), and cause of death (The Cause of Death Register [21]) is also included. A CONSORT flow diagram of the study cohort is presented as Fig. 1. Of the initial 74,994 women, 4885 women responded to the KARMA survey, but did not subsequently register in the study, a further 3163 women did not respond to the survey, leaving 66,946 women in the cohort. For this study, a further 2810 women were excluded due to (i) prevalent breast cancer, (ii) bilateral breast cancer, or lastly to avoid including patients with possible prevalent breast cancer (iii) breast cancer diagnosis or death of any cause within 90 days after baseline, leaving 64,136 individuals out of which 1238 were subsequently diagnosed with incident breast cancer. Finally, 26,197 pre- and perimenopausal women were excluded, leaving 35,412 postmenopausal women in this study, whereof 822 incident breast cancer cases were diagnosed and out of which 726 had full information on all factors used in the adjusted models. All participants signed informed consent and the study was approved by the ethical committee of the Karolinska Institute (# 2017/958).

### Data collection and classification

Data on medications were retrieved from the Swedish Prescription Register [19]. Linkage to the national Swedish Cancer Register [20] was performed to identify all cancer diagnoses, linkage to The Cause of Death Register [21] for causes of death, and linkage to the breast cancer specific NKBC (National Quality Register of Breast Cancer [18]) registers to acquire patient and pathological data for all incident cases including age at diagnosis, tumor size (≤ 20 mm, vs > 20 mm), nodal status (positive/negative), presence of distant metastasis (yes/no), Nottingham Histological Grade (III versus I + II). ER−, PR−, (positive/negative, cutoff > 10% positive cells), and HER2-status (positive/negative), Ki67 (% positive cells, with ≤ 10%, 11–20%, and > 20% defined as low, intermediate, or high). Luminal A was defined by immunohistochemical surrogate markers as ER+HER2− with either (i) histological grade I (irrespective of Ki67), or (ii) histological grade II with low Ki67, or (iii) histological grade II, intermediate Ki67 and PR ≥ 20%. Luminal B was defined as ER+HER2− and either (i) histological grade III (irrespective of Ki67) (ii) histological grade II and high Ki67, or (iii) histological grade II, intermediate Ki67, and PR < 20%.

### Anthropometric data

Self-reported body mass index (BMI) was accessed through the KARMA-questionnaires and divided and analyzed according to the WHO definition into the following groups: Underweight BMI < 18.5 kg/m^2^, normal weight ≥ 18.5–< 25 kg/m^2^, overweight ≥ 25–< 30 kg/m^2^, and obesity BMI ≥ 30 kg/m^2^.

### Co-variates

Data on life-style and reproductive health factors were accessed through the KARMA-questionnaires and included age at menarche, number of pregnancies, parity, age at first childbirth, use of hormonal contraception, hormone replacement therapy, and breast cancer heredity. Life-style factors included smoking and alcohol. Use of co-medications were derived from the Swedish national Prescription Registry including statins (ATC code C10), insulin (ATC code A10A), and metformin (ATC code C10).

### Statistical methods

Participants were followed from date of inclusion in the KARMA study until date of breast cancer diagnosis, date of death, or December 31, 2019, whichever came first. Descriptive statistics on baseline characteristics are presented in Table 1. Descriptive statistics on tumor characteristics for all breast cancer cases (*n* = 822) are presented in Table 2. Participants with missing values for variables adjusted for were excluded from all subsequent analyses. Cumulative incidence of invasive breast cancer with regard to (i) all incident breast cancer and (ii) breast cancer defined by known prognostic variables (defined by TNM, [tumour, node, metastases], age at diagnosis, histological grade, and expression of ER, PR, and HER2) and (iii) subtype-specific breast cancer defined by immunohistochemical surrogate markers with death as a competing risk was calculated using the Aalen-Johansen estimator. Hazard ratios (HR) with 95% confidence intervals (CI) for (i) all incident breast cancer and (ii) breast cancer defined by known prognostic variables and (iii) subtype-specific breast cancer were calculated using Cox proportional hazards model with time on study as the underlying time scale, adjusting for age, age at menarche (continuous), number of pregnancies (continuous), parity (categorical, five levels), age at first child birth (categorical, five levels), use of hormonal contraception (yes/no), hormone replacement therapy (yes/no), breast cancer in family (yes/no), and use of co-medications insulin, metformin, and/or statins (yes/no). Lifestyle factors included smoking (pack years categorical, three levels), and alcohol (yes/no, and grams per week). Age at inclusion, age at menarche, and alcohol were incorporated into the model as natural cubic splines with four knots. The proportionality assumption was checked visually by inspection of the log minus log of the survival curve based on the Kaplan–Meier estimator, and no violation was found.

**Table 1:** Base-line characteristics in relation to BMI in the 35,412 postmenopausal patients in the KARMA Cohort

Variable	Overall	< 18.5	≥ 18.5- < 25	≥ 25- < 30	≥ 30	Missing
No. of women (%)	35 412 (100)	374 (1%)	17 890 (51%)	12 029 (34%)	4693 (13%)	426 (1%)
Age at entry, years (median [IQR])	62 [57, 67]	63 [59, 68]	62 [56, 67]	62 [57, 67]	62 [57, 67]	62 [58, 67]
Age at entry, years (%)						
≤ 29	0 (0.0)	0 (0.0)	0 (0.0)	0 (0.0)	0 (0.0)	0 (0.0)
30–39	17 (0.0)	0 (0.0)	7 (0.0)	7 (0.1)	3 (0.1)	0 (0.0)
40–49	1137 (3.2)	17 (4.5)	623 (3.5)	317 (2.6)	166 (3.5)	14 (3.3)
50–59	12 403 (35.0)	92 (24.6)	6579 (36.8)	4017 (33.4)	1567 (33.4)	148 (34.7)
60–69	17 400 (49.1)	198 (52.9)	8573 (47.9)	6092 (50.6)	2331 (49.7)	206 (48.4)
70–79	4431 (12.5)	67 (17.9)	2094 (11.7)	1588 (13.2)	624 (13.3)	58 (13.6)
≥ 80	24 (0.1)	0 (0.0)	14 (0.1)	8 (0.1)	2 (0.0)	0 (0.0)
Height, cm (median [IQR])	166 [162, 170]	167 [163, 171]	167 [162, 170]	165 [162, 170]	165 [161, 169]	NA [NA, NA]
Weight, kg (median [IQR])	68 [62, 77]	50 [47, 52]	62 [58, 67]	74 [70, 79]	89 [83, 96]	NA [NA, NA]
BMI at entry, kg/m^2^ (median [IQR])	24.8 [22.6, 27.7]	18.0 [17.6, 18.3]	22.7 [21.4, 23.8]	26.9 [25.9, 28.1]	32.3 [30.9, 34.7]	NA [NA, NA]
Age at menarche, years (median [IQR])	13 [12, 14]	13 [12, 14]	13 [12, 14]	13 [12, 14]	13 [12, 14]	13 [12, 14]
Age at menarche, missing (%)	1261 (3.6)	25 (6.7)	495 (2.8)	361 (3.0)	146 (3.1)	234 (54.9)
Age at menopause, years (median [IQR])	50.0 [47.0, 53.0]	50.0 [47.5, 53.0]	50.0 [48.0, 53.0]	50.0 [47.0, 53.0]	50.0 [47.0, 53.0]	50.0 [47.0, 52.3]
Age at menopaus, missing (%)	18 161 (51.3)	203 (54.3)	9042 (50.5)	6146 (51.1)	2408 (51.3)	362 (85.0)
No. of pregnancies (%)						
0	3057 (8.6)	53 (14.2)	1623 (9.1)	915 (7.6)	438 (9.3)	28 (6.6)
1	3934 (11.1)	55 (14.7)	1989 (11.1)	1318 (11.0)	545 (11.6)	27 (6.3)
2	11 371 (32.1)	106 (28.3)	5833 (32.6)	3921 (32.6)	1473 (31.4)	38 (8.9)
3	8656 (24.4)	91 (24.3)	4347 (24.3)	3051 (25.4)	1137 (24.2)	30 (7.0)
≥ 4	7591 (21.4)	58 (15.5)	3853 (21.5)	2636 (21.9)	1012 (21.6)	32 (7.5)
Missing	803 (2.3)	11 (2.9)	245 (1.4)	188 (1.6)	88 (1.9)	271 (63.6)
No. of births (%)						
0	4331 (12.2)	69 (18.4)	2315 (12.9)	1304 (10.8)	607 (12.9)	36 (8.5)
1	5232 (14.8)	73 (19.5)	2619 (14.6)	1784 (14.8)	723 (15.4)	33 (7.7)
2	16,048 (45.3)	134 (35.8)	8211 (45.9)	5625 (46.8)	2025 (43.1)	53 (12.4)
3	7084 (20.0)	71 (19.0)	3605 (20.2)	2442 (20.3)	942 (20.1)	24 (5.6)
≥ 4	1911 (5.4)	16 (4.3)	893 (5.0)	684 (5.7)	309 (6.6)	9 (2.1)
Missing	806 (2.3)	11 (2.9)	247 (1.4)	190 (1.6)	87 (1.9)	271 (63.6)
Age at first birth (%)						
≤ 20	4195 (11.8)	23 (6.1)	1627 (9.1)	1717 (14.3)	805 (17.2)	23 (5.4)
> 20- ≤ 25	11 315 (32.0)	107 (28.6)	5463 (30.5)	4041 (33.6)	1659 (35.4)	45 (10.6)
> 25- ≤ 30	9645 (27.2)	99 (26.5)	5344 (29.9)	3163 (26.3)	1006 (21.4)	33 (7.7)
> 30	5107 (14.4)	65 (17.4)	2887 (16.1)	1611 (13.4)	526 (11.2)	18 (4.2)
Nulliparous	4331 (12.2)	69 (18.4)	2315 (12.9)	1304 (10.8)	607 (12.9)	36 (8.5)
Missing	819 (2.3)	11 (2.9)	254 (1.4)	193 (1.6)	90 (1.9)	271 (63.6)
Age at first child birth, years (median [IQR])	25.0 [22.0, 29.0]	26.0 [23.0, 30.0]	26.0 [23.0, 29.0]	25.0 [22.0, 28.0]	24.0 [21.0, 28.0]	25.0 [21.0, 28.0]
No. of women using oral contraceptives (%)						
No	6701 (18.9)	91 (24.3)	3241 (18.1)	2304 (19.2)	1031 (22.0)	34 ( 8.0)
Yes	27 308 (77.1)	266 (71.1)	14 101 (78.8)	9323 (77.5)	3494 (74.5)	124 (29.1)
Missing	1403 (4.0)	17 (4.5)	548 (3.1)	402 (3.3)	168 (3.6)	268 (62.9)
No. of women using HRT (%)						
No	19 759 (55.8)	209 (55.9)	9871 (55.2)	6715 (55.8)	2833 (60.4)	131 (30.8)
Yes	14 797 (41.8)	152 (40.6)	7740 (43.3)	5085 (42.3)	1754 (37.4)	66 (15.5)
Missing	856 (2.4)	13 (3.5)	279 (1.6)	229 (1.9)	106 (2.3)	229 (53.8)
No. of women with breast cancer in the family (%)						
No	28 789 (81.3)	301 (80.5)	14 738 (82.4)	9822 (81.7)	3766 (80.2)	162 (38.0)
Yes	5250 (14.8)	60 (16.0)	2644 (14.8)	1788 (14.9)	735 (15.7)	23 (5.4)
Missing	1373 (3.9)	13 (3.5)	508 (2.8)	419 (3.5)	192 (4.1)	241 (56.6)
No. of smoking women (%)						
Never	14 172 (40.0)	163 (43.6)	7486 (41.8)	4634 (38.5)	1830 (39.0)	59 (13.8)
Previous	16 026 (45.3)	118 (31.6)	7885 (44.1)	5726 (47.6)	2233 (47.6)	64 (15.0)
Current	4342 (12.3)	83 (22.2)	2234 (12.5)	1452 (12.1)	544 (11.6)	29 (6.8)
Missing	872 (2.5)	10 (2.7)	285 (1.6)	217 (1.8)	86 (1.8)	274 (64.3)
Smoking, packyears (median [IQR])	2.00 [0.00, 10.5]	0.90 [0.00, 14.2]	1.50 [0.00, 8.60]	2.80 [0.00, 11.8]	3.90 [0.00, 14.3]	3.45 [0.00, 11.8]
No. of women drinking alcohol (%)						
No	6619 (18.7)	88 (23.5)	2789 (15.6)	2224 (18.5)	1475 (31.4)	43 (10.1)
Yes	27 686 (78.2)	273 (73.0)	14 707 (82.2)	9515 (79.1)	3084 (65.7)	107 (25.1)
Missing	1107 (3.1)	13 (3.5)	394 (2.2)	290 (2.4)	134 (2.9)	276 (64.8)
Alcohol, gram per week (median [IQR])	37.0 [6.00, 67.0]	36.0 [5.00, 68.0]	37.0 [12.0, 68.00]	37.0 [6.00, 68.0]	24.0 [0.00, 49.0]	24.5 [0.00, 48.8]
No. of women using statins (%)						
No	30 742 (86.8)	349 (93.3)	16 186 (90.5)	10 152 (84.4)	3684 (78.5)	371 (87.1)
Yes	4670 (13.2)	25 (6.7)	1704 (9.5)	1877 (15.6)	1009 (21.5)	55 (12.9)
No. of women using insulin (%)						
No	34 985 (98.8)	368 (98.4)	17 774 (99.4)	11 908 (99.0)	4514 (96.2)	421 (98.8)
Yes	427 (1.2)	6 (1.6)	116 (0.6)	121 (1.0)	179 (3.8)	5 (1.2)
No. of women using metformin (%)						
No	34 563 (97.6)	374 (100.0)	17 757 (99.3)	11 735 (97.6)	4283 (91.3)	414 (97.2)
Yes	849 (2.4)	0 (0.0)	133 (0.7)	294 (2.4)	410 (8.7)	12 (2.8)

**Table 2:** Patient- and tumor characteristics of the 822 women diagnosed with an incident breast cancer

Variable	Levels	Postmenopausal patients
Overall		822
Age at diagnosis (median (IQR))		68.0 (63.0, 71.0)
BMI at baseline, kg/m2 (median (IQR))		25.1 (22.9, 27.7)
Tumor size, mm (median (IQR))		14.0 (10.0, 20.0)
Tumor size (No., %)	T0	1 (0.1)
	T1 (1–20 mm)	537 (65.3)
	T2 (21–50 mm)	164 (20.0)
	T3 (> 50 mm)	27 (3.3)
	T4	1 (0.1)
	Missing	92 (11.2)
Nodal status (No., %)	Negative	529 (64.4)
	Positive	201 (24.5)
	Missing	92 (11.2)
Distant metastases at diagnosis (No., %)	Negative	738 (89.8)
	Positive	7 (0.9)
	Missing	77 (9.4)
ER status (No., %)	Negative	80 (9.7)
	Positive	642 (78.1)
	Missing	100 (12.2)
PR status (No., %)	Negative	222 (27.0)
	Positive	493 (60.0)
	Missing	107 (13.0)
HER2 status (No., %)	Negative	645 (78.5)
	Positive	81 (9.9)
	Missing	96 (11.7)
Histological grade (No., %)	1	159 (19.3)
	2	348 (42.3)
	3	188 (22.9)
	Missing	127 (15.5)
Ki67 (No., %)	High	315 (38.3)
	Intermediate	107 (13.0)
	Low	283 (34.4)
	Missing	117 (14.2)
ER+/HER2− (No., %)	No	137 (16.7)
	Yes	577 (70.2)
	Missing	108 (13.1)
ER+/HER2+ (No., %)	No	656 (79.8)
	Yes	58 (7.1)
	Missing	108 (13.1)
ER−/HER2+ (No., %)	No	695 (84.5)
	Yes	19 (2.3)
	Missing	108 (13.1)
TNBC (No., %)	No	654 (79.6)
	Yes	60 (7.3)
	Missing	108 (13.1)
Luminal A—like (No., %)	No	301 (36.6)
	Yes	351 (42.7)
	Missing	170 (20.7)
Luminal B—like (No., %)	No	472 (57.4)
	Yes	187 (22.7)
	Missing	163 (19.8)

## Results

### Patient and tumor characteristics

Detailed information on the baseline characteristics of all 35,412 participants are presented in Table 1. Median age at baseline was 62 years (Inter Quartile Range; IQR 57–67). Median BMI 24.8 kg/m^2^ (IQR 22.6–27.7) and 12,029 (34%) of the population was defined as overweight and 4693 (13%) as obese.

Table 2 presents patient- and tumor characteristics in the 822 breast cancer patients. The median age at diagnosis was 68.0 years (IQR 63.0–71.0), median BMI 25.1 kg/m^2^ (IQR 22.9–27.7), median tumor size 14.0 mm (IQR 10.0–20.0). At the time of diagnosis, 24.5% were lymph node positive, 0.9% had distant metastases, 78.1% were ER+, 60.0% PR+, 9.9% HER2+, 22.9% with histological grade III, and 38.3% had tumors with high Ki67. Based on immunohistochemical surrogate markers for subtyping, 70.2% were luminal-like (ER+/HER2−) out of which 42.7% were Luminal-A-like and 22.7% Luminal-B-like. Another 7.1% were ER+/HER2+, 2.3% ER−/HER2+, and lastly, 7.3% were diagnosed with TNBC.

### BMI and risk of breast cancer

The median follow-up time was 2719 days (7.4 years). Tables 3 displays the risk of breast cancer in relation to BMI. There was an increased risk of breast cancer among overweight women compared with normal-weight women (crude HR 1.20, 95% CI 1.02–1.40), which remained significant after adjusting for age at menarche, use of HRT and oral contraceptives, age at first child birth, number of births, co-medications (insulin, metformin, and statins), heredity, and life-style factors (smoking and alcohol) (HR_adj_ 1.19, 95% CI 1.01–1.40). A similar, however, not significant, association was found among obese women (crude HR 1.14, 95% CI 0.91–1.43, HR_adj_ 1.19, 95% CI 0.94–1.50, respectively).

**Table 3:** Crude rates per 1000 person years, 8-year cumulative risk, crude and adjusted* hazard ratios for breast cancer in relation to BMI

	Persons	Cases	Person years	Crude rate per 1000 person years (95% CI)	8-year cumulative risk (95% CI)	Crude HR (95% CI)	Adjusted HR (95% CI)
BMI							
< 18.5	320	5	2260	2.21 (0.72–5.16)	1.56% (0.59%-3.43%)	0.76 (0.31–1.83)	0.72 (0.30–1.74)
≥ 18.5– < 25	16 233	346	119 000	2.91 (2.61–3.24)	2.23% (1.99%-2.48%)	1 (reference)	1 (reference)
≥ 25– < 30	10 809	275	78 800	3.49 (3.09–3.93)	2.61% (2.31%-2.93%)	1.20 (1.02–1.40)	1.19 (1.01–1.40)
≥ 30	4148	100	30 000	3.33 (2.71–4.05)	2.48% (2.03%-3.00%)	1.14 (0.91–1.43)	1.19 (0.94–1.50)

*Adjusted for reproductive factors (age at menarche, use of HRT and oral contraceptives, age at first child birth, number of births, co-medications (insulin, metformin, and statins), heredity, and life-style factors (smoking and alcohol)

### BMI and risk in relation to known prognostic variables and subtype-specific breast cancer

Table 4 displays the risk of breast cancer based on prognostic factors in relation to BMI. During follow-up, there was an increased risk of ER+ breast cancer among the overweight (HR_adj_ 1.20, 95% CI 1.00–1.44), and obese women (HR_adj_ 1.33, 95% CI 1.03–1.71), compared with normal-weight women. Similarly, there was an increased risk of PR+ breast cancer in obese women only (HR_adj_ 1.53, 95% CI 1.16–2.02), with a similar but not significant association for overweight women. There was also an association with being diagnosed with low-grade tumors in overweight (HR_adj_ 1.2, 95% CI 1.02–1.54), and obese women (HR_adj_ 1.40, 95% CI 1.05–1.86). A similar but non-significant association was found for tumors with low Ki67. According to node status, there was an increased risk of node-positive disease in obese women (HR_adj_ 1.64, 95% CI 1.09–2.48). For overweight women there was instead an increased risk of node-negative disease (HR_adj_ 1.29, 95% CI 1.06–1.58). No significant association with either overweight or obesity were found for the other prognostic factors, such as tumor size or HER2-status.

**Table 4:** Crude rates per 1000 person years, 8-year cumulative risk, and crude and adjusted* hazard ratios for known prognostic breast cancer variables in relation to BMI

	Postmenopausal patients							
Variable	BMI	Persons	Cases	Person years	Crude rate per 1000 person years (95% CI)	8-year cumulative risk (95% CI)	Crude HR (95% CI)	Adjusted HR (95% CI)
Tumor size < 20 mm	< 25	16 553	228	121 000	1.88 (1.65–2.15)	1.46% (1.27%-1.67%)	1 (reference)	1 (reference)
	≥ 25- < 30	10 809	178	78 800	2.26 (1.94–2.62)	1.70% (1.46%-1.97%)	1.20 (0.99–1.46)	1.20 (0.98–1.46)
	≥ 30	4148	64	30 000	2.13 (1.64–2.72)	1.63% (1.27%-2.08%)	1.13 (0.86–1.49)	1.20 (0.90–1.60)
Tumor size > 20 mm	< 25	16 553	78	121 000	0.65 (0.51–0.81)	0.50% (0.39%-0.62%)	1 (reference)	1 (reference)
	≥ 25- < 30	10 809	63	78 800	0.80 (0.62–1.02)	0.62% (0.48%-0.79%)	1.24 (0.90–1.73)	1.23 (0.88–1.72)
	≥ 30	4148	27	30 000	0.90 (0.59–1.31)	0.66% (0.45%-0.95%)	1.39 (0.90–2.16)	1.37 (0.87–2.16)
Nodal status Negative	< 25	16 553	218	121 000	1.80 (1.57–2.06)	1.40% (1.21%-1.60%)	1 (reference)	1 (reference)
	≥ 25- < 30	10 809	184	78 800	2.34 (2.01–2.70)	1.77% (1.52%-2.04%)	1.30 (1.06–1.58)	1.29 (1.06–1.58)
	≥ 30	4148	58	30 000	1.93 (1.47–2.50)	1.47% (1.12%-1.89%)	1.07 (0.80–1.43)	1.12 (0.83–1.51)
Nodal status Positive	< 25	16 553	89	121 000	0.74 (0.59–0.91)	0.57% (0.45%-0.71%)	1 (reference)	1 (reference)
	≥ 25- < 30	10 809	57	78 800	0.72 (0.55–0.94)	0.55% (0.42%-0.71%)	0.98 (0.71–1.37)	0.98 (0.70–1.37)
	≥ 30	4148	35	30 000	1.17 (0.81–1.62)	0.88% (0.62%-1.21%)	1.58 (1.07–2.34)	1.64 (1.09–2.48)
ER status Positive	< 25	16 553	269	121 000	2.22 (1.97–2.51)	1.71% (1.51%-1.93%)	1 (reference)	1 (reference)
	≥ 25- < 30	10 809	211	78 800	2.68 (2.33–3.07)	2.03% (1.76%-2.32%)	1.20 (1.01–1.44)	1.20 (1.00–1.44)
	≥ 30	4148	85	30 000	2.83 (2.26–3.5)	2.11% (1.70%-2.59%)	1.27 (1.00–1.62)	1.33 (1.03–1.71)
ER status Negative	< 25	16 553	37	121 000	0.31 (0.22–0.42)	0.25% (0.17%-0.35%)	1 (reference)	1 (reference)
	≥ 25- < 30	10 809	24	78 800	0.31 (0.20–0.45)	0.24% (0.16%-0.36%)	1.00 (0.60–1.66)	0.99 (0.59–1.67)
	≥ 30	4148	7	30 000	0.23 (0.094–0.48)	0.21% (0.09%-0.44%)	0.76 (0.34–1.71)	0.79 (0.34–1.81)
PR status Positive	< 25	16 553	204	121 000	1.69 (1.46–1.93)	1.31% (1.13%-1.51%)	1 (reference)	1 (reference)
	≥ 25- < 30	10 809	162	78 800	2.06 (1.75–2.40)	1.56% (1.33%-1.82%)	1.22 (0.99–1.50)	1.21 (0.99–1.50)
	≥ 30	4148	75	30 000	2.50 (1.96–3.13)	1.86% (1.48%-2.32%)	1.48 (1.14–1.93)	1.53 (1.16–2.02)
PR status Negative	< 25	16 553	99	121 000	0.82 (0.67–1.00)	0.63% (0.51%-0.77%)	1 (reference)	1 (reference)
	≥ 25- < 30	10 809	70	78 800	0.89 (0.69–1.12)	0.68% (0.53%-0.86%)	1.08 (0.80–1.47)	1.09 (0.80–1.48)
	≥ 30	4148	17	30 000	0.57 (0.33–0.91)	0.46% (0.27%-0.73%)	0.69 (0.41–1.16)	0.74 (0.44–1.25)
HER2 status Negative	< 25	16 553	272	121 000	2.25 (1.99–2.53)	1.75% (1.54%-1.97%)	1 (reference)	1 (reference)
	≥ 25- < 30	10 809	209	78 800	2.65 (2.31–3.04)	2.01% (1.75%-2.30%)	1.18 (0.99–1.41)	1.18 (0.98–1.41)
	≥ 30	4148	83	30 000	2.76 (2.20–3.43)	2.10% (1.68%-2.59%)	1.23 (0.96–1.57)	1.29 (1.00–1.66)
HER2 status Positive	< 25	16 553	34	121 000	0.28 (0.20–0.39)	0.21% (0.15%-0.29%)	1 (reference)	1 (reference)
	≥ 25- < 30	10 809	29	78 800	0.37 (0.25–0.53)	0.28% (0.19%-0.40%)	1.31 (0.80–2.15)	1.32 (0.80–2.18)
	≥ 30	4148	10	30 000	0.33 (0.16–0.61)	0.24% (0.13%-0.44%)	1.18 (0.59–2.39)	1.22 (0.59–2.53)
Histological grade 1/2	< 25	16 553	209	121 000	1.73 (1.50–1.98)	1.33% (1.15%-1.54%)	1 (reference)	1 (reference)
	≥ 25- < 30	10 809	171	78 800	2.17 (1.86–2.52)	1.65% (1.42%-1.92%)	1.26 (1.03–1.54)	1.25 (1.02–1.54)
	≥ 30	4148	67	30 000	2.23 (1.73–2.83)	1.64% (1.28%-2.07%)	1.29 (0.98–1.70)	1.40 (1.05–1.86)
Histological grade 3	< 25	16 553	82	121 000	0.68 (0.54–0.84)	0.53% (0.42%-0.66%)	1 (reference)	1 (reference)
	≥ 25- < 30	10 809	58	78 800	0.74 (0.60–0.95)	0.55% (0.43%-0.71%)	1.09 (0.78–1.52)	1.06 (0.75–1.49)
	≥ 30	4148	20	30 000	0.67 (0.41–1.03)	0.56% (0.35%-0.86%)	0.98 (0.60–1.60)	0.92 (0.56–1.54)
Ki67 Low	< 25	16 553	161	121 000	1.33 (1.13–1.55)	1.01% (0.86%-1.19%)	1 (reference)	1 (reference)
	≥ 25- < 30	10 809	131	78 800	1.66 (1.39–1.97)	1.28% (1.07%-1.53%)	1.25 (0.99–1.57)	1.26 (0.99–1.59)
	≥ 30	4148	51	30 000	1.70 (1.26–2.23)	1.25% (0.94%-1.63%)	1.28 (0.93–1.75)	1.37 (0.99–1.9)
Ki67 High	< 25	16 553	138	121 000	1.14 (0.96–1.35)	0.90% (0.75%-1.07%)	1 (reference)	1 (reference)
	≥ 25- < 30	10 809	99	78 800	1.26 (1.02–1.53)	0.94% (0.76%-1.14%)	1.10 (0.85–1.42)	1.1 (0.85–1.43)
	≥ 30	4148	39	30 000	1.30 (0.92–1.77)	1.02% (0.73%-1.40%)	1.14 (0.80–1.62)	1.16 (0.80–1.67)

*Adjusted for reproductive factors (age at menarche, use of HRT and oral contraceptives, age at first child birth, number of births, co-medications (insulin, metformin, and statins), heredity, and life-style factors (smoking and alcohol)

Lastly, in Table 5 the risk of subtype-specific breast cancer, based on immunohistochemical surrogate markers for subtype, and BMI is displayed. There was an increased risk of luminal ER+HER− breast cancer in obese women (crude HR 1.29, 95% CI 1.00–1.67, and HR_adj_ 1.37, 95% CI 1.05–1.78, respectively), with a similar but non-significant association in overweight women. There was also an association with risk of low-proliferative Luminal A cancers among obese women (crude HR 1.34 (95% CI 0.97–1.86) and HR_adj_ 1.43 (95% CI 1.02–2.01), respectively). No associations were found for either TNBC, high-proliferative Luminal B tumors, or HER2+ tumors, and either overweight or obesity.

**Table 5:** Crude rates per 1000 person years, 8-year cumulative risk, crude and adjusted* hazard ratios for immunohistochemical surrogate marker subtype specific breast cancer in relation to BMI

	Postmenopausal patients							
Subtype	BMI	Persons	Cases	Person years	Crude rate per 1000 person years (95% CI)	8-year cumulative risk (95% CI)	Crude HR (95% CI)	Adjusted HR (95% CI)
ER+, HER2−	< 25	16 553	243	121 000	2.01 (1.76–2.28)	1.55% (1.35–1.76%)	1 (reference)	1 (reference)
	≥ 25–< 30	10 809	185	78 800	2.35 (2.02–2.71)	1.77% (1.53–2.05%)	1.17 (0.97–1.41)	1.17 (0.96–1.42)
	≥ 30	4148	78	30 000	2.6 (2.05–3.24)	1.94% (1.54–2.40%)	1.29 (1.00–1.67)	1.37 (1.05–1.78)
ER+, HER2+	< 25	16 553	22	121 000	0,18 (0.11–0.28)	0.14% (0.09–0.21%)	1 (reference)	1 (reference)
	≥ 25–< 30	10 809	23	78 800	0.29 (0.19–0.44)	0.22% (0.15–0.33%)	1.60 (0.89–2.88)	1.57 (0.87–2.83)
	≥ 30	4148	7	30 000	0.23 (0.094–0.48)	0.17% (0.08–0.34%)	1.28 (0.55–3.00)	1.19 (0.50–2.88)
ER−, HER2+	< 25	16 553	11	121 000	0.091 (0.045–0.16)	0.07% (0.04–0.12%)	1 (reference)	1 (reference)
	≥ 25–< 30	10 809	3	78 800	0.038 (0.0079–0.11)	0.03% (0.01–0.08%)	0.42 (0.12–1.50)	0.46 (0.13–1.65)
	≥ 30	4148	3	30 000	0.10 (0.021–0.29)	0.07% (0.02–0.21%)	1.09 (0.31–3.92)	1.35 (0.36–5.01)
TNBC	< 25	16 553	26	121 000	0.22 (0.14–0.32)	0.18% (0.12–0.27%)	1 (reference)	1 (reference)
	≥ 25–< 30	10 809	21	78 800	0.27 (0.17–0.41)	0.21% (0.13–0.32%)	1.24 (0.70–2.21)	1.22 (0.68–2.19)
	≥ 30	4148	4	30 000	0.13 (0.036–0.34)	0.14% (0.05–0.36%)	0.62 (0.22–1.78)	0.61 (0.21–1.79)
Luminal A	< 25	16 553	144	121 000	1.19 (1.00–1.40)	0.86% (0.72–1.01%)	1 (reference)	1 (reference)
	≥ 25–< 30	10 809	116	78 800	1.47 (1.22–1.77)	1.08% (0.90–1.29%)	1.24 (0.97–1.58)	1.23 (0.96–1.57)
	≥ 30	4148	48	30 000	1.6 (1.18–2.12)	1.16% (0.87–1.53%)	1.34 (0.97–1.86)	1.43 (1.02–2.01)
Luminal B	< 25	16 553	79	121 000	0.65 (0.52–0.81)	0.52% (0.41–0.66%)	1 (reference)	1 (reference)
	≥ 25–< 30	10 809	60	78 800	0.76 (0.58–0.98)	0.56% (0.43–0.72%)	1.17 (0.83–1.63)	1.15 (0.82–1.62)
	≥ 30	4148	26	30 000	0.87 (0.57–1.27)	0.67% (0.44–0.97%)	1.32 (0.85–2.06)	1.33 (0.84–2.11)

*Adjusted for reproductive factors (age at menarche, use of HRT and oral contraceptives, age at first child birth, number of births, co-medications (insulin, metformin, and statins), heredity, and life-style factors (smoking and alcohol)

## Discussion

In this large, contemporary prospective Swedish cohort of postmenopausal women included during modern screening time period (2011–2013), we found an association between overweight and breast cancer risk. Our findings validate previous studies on the association between body weight and risk of breast cancer [4–7]. Here we show that the associations are specifically relevant for ER+, low-grade breast cancer among overweight women, whereas in obese women there is an increased risk of node-positive breast cancer.

Overweight in postmenopausal women has in previous studies not only been associated with an increased risk of developing breast cancer, especially ER+ breast cancer, but also with a worse prognosis [2, 22]. Studies have also found that weight-loss, including by means of bariatric surgery, reduces the risk of breast cancer [23, 24] and may also improve breast cancer outcome [5, 25].

The mechanisms underlying the increased risks of obese women in developing postmenopausal receptor positive breast cancer are multi-factorial and mainly linked to hormonal pathways [26]. Overweight women have higher circulating levels of estrogen due to increased expression of aromatase in the adipose tissue [27–29]. Excess weight is also associated with high levels of insulin and insulin-like growth factor-I levels, which are mitogenic [28, 30]. Insulin also inhibits sex hormone-binding globulin levels [31], leading to higher levels of biologically active estrogens [32], which in turn can induce tumor cell proliferation and inhibit apoptosis [31]. Other obesity-associated factors affecting the risk of breast cancer are increases in levels of pro-inflammatory cytokines and leptin, which increases aromatization, and decreased levels of the anti-inflammatory and insulin-sensitizing adiponectin [31]. As receptor-negative tumors are less dependent on estrogen, this may explain the weaker association with overweight and development of ER-negative tumors, even though preclinical studies have suggested that obesity might promote TNBCs through insulin resistance, secretion of pro-angiogenic adipokines such as leptin, and chronic inflammation [33].

The impact of overweight or weight-gain on the risk of developing breast cancer may also vary over a lifetime [34, 35]. A recent meta-analysis found a strong positive and non-linear association between BMI and postmenopausal, receptor positive breast cancer, especially in women who had not used HRT [29]. For women with overweight in early adulthood, there was instead a reduced risk of postmenopausal breast cancer, independent of later weight-gain. For women who did gain weight after early adulthood, especially leaner women, there was instead an increased risk of receptor positive breast cancer [4, 29], which has been further validated in a recent Mendelian Randomization study [8]. Some studies have also suggested that the positive association between obesity and postmenopausal breast cancer is more pronounced in older postmenopausal women. As the present cohort consists mainly of patients of mammography screening age, 40 to 74 years, had an average age at inclusion of 62 years and a mean follow-up of 7.4 years, the association might become stronger as follow-up time increases.

In our study, we found an association between BMI and breast cancer risk in the overweight group, with a similar but not statistically significant risk among the obese participants. Although many studies find a linear association with the risk of breast cancer increasing with BMI, our results are instead in line with a recent meta-analysis, which found a strong positive but non-linear association between BMI and postmenopausal, receptor positive breast cancer, especially in women who had not used HRT [29]. In that meta-analysis, they found an upper threshold for the effect of BMI above 28 or 30 kg/m^2^ after which the risk of breast cancer did not increase [29]. The biological explanation for the threshold effect is unclear but may be explained by ER-mediated effects.

In line with previous publications, we found a positive association between both overweight and obesity and risk of ER+ breast cancer, low-grade breast cancer, and with a significant association between obesity and risk of ER+/HER2− and luminal A tumors [5, 9, 36]. Even though preclinical studies have suggested that obesity might promote TNBC through chronic inflammation, insulin resistance, and secretion of pro-angiogenic adiopkines [33], results in clinical studies are conflicting [4, 5, 12, 14]. We found no associations with overweight or obesity and ER- or TNBC, which may be difficult to interpret due to low numbers in the present study. As for other established prognostic factors and BMI there was an increased risk of node-positive breast cancer in the obese, but not the overweight, women.

The strengths of the study population are the prospective set-up of a contemporary cohort, representing breast cancer diagnoses of today under the influence of the rising overweight/obesity prevalence with availability of extensive questionnaires with data on BMI and confounders at time of inclusion. The limitations are the relative low number of cases, multiple comparisons, the follow-up of 7.4 years, and the low mean age as the relationship between adiposity and breast cancer risk is more pronounced in older women. With longer follow-up and more cases more pronounced associations would be expected. Lastly, as molecular subtyping was not part of the routine pathological diagnostic procedures at the time of inclusion in the present study, analysis of subtypes relies on immunohistochemical assessments rather than molecular subtyping.

In conclusion this study finds overweight and obesity to be associated with an increased risk of developing breast cancer, specifically ER+, low-grade, and for obesity, node-positive, high-risk breast cancer. As overweight is an increasing global health problem and is also one of few modifiable cancer risk factors, with studies finding that weight-loss reduces the risk of breast cancer and may also improve breast cancer outcome, risk communication, and weight-control will remain an important intervention in reducing the incidence and improving the prognosis of postmenopausal breast cancer. 

## Data Availability

The datasets generated during and/or analyzed during the current study are not publicly available due to GDPR regulations, but full de-identifiable data are available from the corresponding author on reasonable request.
